# Supplementary material for: Steroid‐dependent switch of OvoL/Shavenbaby controls self‐renewal versus differentiation of intestinal stem cells
Source: EMBO J. 2020 Dec 29;40(4):e104347. doi: 10.15252/embj.2019104347 (PMC7883054; doi:10.15252/embj.2019104347)
Supplement: Supplementary file 1 — Appendix [file EMBJ-40-e104347-s001.pdf]

# Steroid-dependent switch of OvoL/ Shavenbaby controls self-renewal versus differentiation of intestinal stem cells

Al Hayek *et al.*,

## APPENDIX

---

Appendix Table S1

This file contains a full description of the genotypes, as they appear in each panel of main and extended view figures.

| |
|---|
| **Figure 1** |
| C) *y, w; esg-LacZ/+; svb-E3N-GFP/+* |
| D) ***control:*** *esg-Gal4, UAS-GFP.nls, tubP-Gal80$^{ts}$/+; Delta-LacZ/+*<br>***svb-IR*:** *esg-Gal4, UAS-GFP.nls, tubP-Gal80$^{ts}$/+; Delta-LacZ/UAS-svb$^{RNAi}$* |
| D') ***control*:** *Su(H)-GBE-LacZ/+; esg-Gal4, tubP-Gal80$^{ts}$/+; UAS-mCherry/+*<br>***svb-IR:*** *Su(H)-GBE-LacZ/+; esg-Gal4, tubP-Gal80$^{ts}$/+; UAS-mCherry/UAS-svb$^{RNAi}$* |
| F) ***control:*** *y, w, hs-FLP, tubP-Gal80, FR19A; UAS-mCD8::GFP/+; tubulin-Gal4/ry$^{506}$*<br>***svb$^{null}$* :** *y, w, hs-FLP, tubP-Gal80, FR19A/ y, w, svb$^{R9}$, FRT19A; UAS-mCD8::GFP/+; tubulin-Gal4/+* |
| G) ***control*:** *esg-Gal4, UAS-GFP, tubP-Gal80$^{ts}$/+; UAS-FLP, act>CD2>Gal4/+*<br>***svb-IR*:** *esg-Gal4, UAS-GFP, tubP-Gal80$^{ts}$/+; UAS-FLP, act>CD2>Gal4/UAS-svb$^{RNAi}$* |
| **Figure 2** |
| B) ***esg-LacZ***: *y, w; esg-LacZ/+*<br>***priA-LacZ***: *w; ; priA-lacZ/priA-LacZ*<br>***priH-LacZ***: *w; ; priH-lacZ/priH-LacZ*<br>***priJ-LacZ***: *w; ; priJ-lacZ/priJ-LacZ*<br>***pri-Gal4>GFP***: *UAS-GFP; pri-Gal4/+* |
| D) ***control***: *esg-Gal4, UAS-mDC8-GFP, tubP-Gal80$^{ts}$/+*<br>***pri-IR***: *esg-Gal4, UAS-mDC8-GFP, tubP-Gal80$^{ts}$/+; UAS-pri$^{RNAi}$/+* |
| E) ***control***: *esg-Gal4, UAS-YFP/+; Su(H)-GBE-Gal80, tubP-Gal80$^{ts}$/+*<br>***EcR-IR$^{\#1}$***: *esg-Gal4, UAS-YFP/UAS-EcR$^{RNAi,BL\#58286}$; Su(H)-GBE-Gal80, tubP-Gal80$^{ts}$/+*<br>***EcR-IR$^{\#2}$***: *esg-Gal4, UAS-YFP/+; Su(H)-GBE-Gal80, tubP-Gal80$^{ts}$/ UAS-EcR$^{RNAi, BL\#2937}$* |
| F) ***control***: *esg-Gal4, UAS-mDC8-GFP, tubP-Gal80$^{ts}$/+;*<br>***EcR-DN***: *esg-Gal4, UAS-mDC8-GFP, tubP-Gal80$^{ts}$/UAS-EcRDN$^{B2w650A}$*<br>***Pri***: *esg-Gal4, UAS-mDC8-GFP, tubP-Gal80$^{ts}$/+; UAS-pri/+*<br>***EcR-DN+Pri***: *esg-Gal4, UAS-mDC8-GFP, tubP-Gal80$^{ts}$/UAS-EcRDN$^{B2w650A}$; UAS-pri/+* |
| G) ***control***: *y, w, hs-FLP, tubP-Gal80, FR19A; UAS-mCD8::GFP/+; tubulin-Gal4/ry$^{506}$*<br>***Ubr3$^{null}$***: *y, w, hs-FLP, tubP-Gal80, FR19A/ y, w, Ubr3$^{B,}$ FRT19A; UAS-mCD8::GFP/+; tubulin-Gal4/+* |
| H) ***control***: *esg-Gal4, UAS-mDC8-GFP, tubP-Gal80$^{ts}$/+;*<br>***Ubr3-IR***: *esg-Gal4, UAS-mDC8-GFP, tubP-Gal80$^{ts}$/UAS-Ubr3$^{RNAi}$*<br>***Ubr3-IR+Svb$^{ACT}$***: *esg-Gal4, UAS-mDC8-GFP, tubP-Gal80$^{ts}$/UAS-Ubr3$^{RNAi}$; UAS-OvoB/+* |
| **Figure 3** |
| A) ***control***: *esg-Gal4, UAS-GFP.nls, tubP-Gal80$^{ts}$/+; Delta-LacZ/+*<br>***Svb$^{ACT}$***: *esg-Gal4, UAS-GFP.nls, tubP-Gal80$^{ts}$/+; Delta-LacZ/UAS-OvoB* |
| B) ***control***: *esg-Gal4, UAS-mDC8-GFP, tubP-Gal80$^{ts}$/+;*<br>***Svb$^{ACT}$***: *esg-Gal4, UAS-mCD8::GFP, tubP-Gal80$^{ts}$/+; UAS-OvoB/+*<br>***svb-IR***: *esg-Gal4, UAS-GFP, tubP-Gal80$^{ts}$/+; UAS-FLP, act>CD2>Gal4/UAS-svb$^{RNAi}$* |
| C) ***control***: *esg-Gal4, UAS-mDC8-GFP, tubP-Gal80$^{ts}$/+;*<br>***Svb$^{ACT}$***: *esg-Gal4, UAS-mDC8-GFP, tubP-Gal80$^{ts}$/UAS-Svb$^{ACT::GFP}$* |
| D) ***miR8***: *UAS-miR8/+ ; esg-Gal4, UAS-mDC8-GFP, tubP-Gal80$^{ts}$/+;*<br>***miR8 + Svb$^{ACT}$***: *UAS-miR8/+ ; esg-Gal4, UAS-mDC8-GFP, tubP-Gal80$^{ts}$/+; UAS-OvoB/+*<br>***Svb$^{ACT}$***: *esg-Gal4, UAS-mCD8::GFP, tubP-Gal80$^{ts}$/+; UAS-OvoB/+* |
| E) ***control***: *esg-Gal4, UAS-mCD8::GFP, tubP-Gal80$^{ts}$/+;*<br>***NICD***: *esg-Gal4, UAS-mCD8::GFP, tubP-Gal80$^{ts}$/+; UAS-NICD/+*<br>***NICD+Svb$^{ACT}$***: *esg-Gal4, UAS-mCD8::GFP, tubP-Gal80$^{ts}$; UAS-NICD/UAS-OvoB* |

## Figure 4

A) ***control***: *esg-Gal4, UAS-GFP, tubP-Gal80*$^{ts}$*/+; UAS-FLP, act>CD2>Gal4/+*
***RasV***$^{12}$: *esg-Gal4, UAS-GFP, tubP-Gal80*$^{ts}$*/UAS-Ras-V12; UAS-FLP, act>CD2>Gal4/+*
***RasV***$^{12}$***+svb-IR***: *esg-Gal4, UAS-GFP, tubP-Gal80*$^{ts}$*/UAS-RasV*$^{12}$*; UAS-FLP, act>CD2>Gal4/UAS-svb*$^{RNAi}$
***control:*** *esg-Gal4, UAS-mDC8::GFP, tubP-Gal80*$^{ts}$*/+;*
***EGFR-DN:*** *esg-Gal4, UAS-mDC8::GFP, tubP-Gal80*$^{ts}$*/UAS-EGFR-DN; UAS-EGFR-DN/+*
***EGFR-DN+Svb***$^{ACT}$***:*** *esg-Gal4, UAS-mDC8::GFP, tubP-Gal80*$^{ts}$*/UAS-EGFR-DN; UAS-OvoB/UAS-EGFR-DN*

B) ***control***: *esg-Gal4, UAS-mDC8::GFP, tubP-Gal80*$^{ts}$*/+;*
***Arm***$^{s10}$: *UAS-Arm*$^{s10}$*/+; esg-Gal4, UAS-mDC8::GFP, tubP-Gal80*$^{ts}$*/+;*
***Arm***$^{s10}$***+svb-IR***: *UAS-Arm*$^{s10}$*/+; esg-Gal4, UAS-mDC8::GFP, tubP-Gal80*$^{ts}$*/+; UAS-svb*$^{RNAi}$*/+*
***Arm***$^{s10}$***+OvoB***: *UAS-Arm*$^{s10}$*/+; esg-Gal4, UAS-mDC8::GFP, tubP-Gal80*$^{ts}$*/+; UAS-OvoB/+*
***TCF-DN***: *esg-Gal4, UAS-mDC8::GFP, tubP-Gal80*$^{ts}$*/UAS-TCF-DN;*
***TCF-DN+Svb***$^{ACT}$: *esg-Gal4, UAS-mDC8::GFP, tubP-Gal80*$^{ts}$*/UAS-TCF-DN; UAS-OvoB/+*

C) ***E3N-wt***: *w; E3N-LacZ/E3N-LacZ*
***E3N-Pnt-mt***: *w; E3N-Pnt-mt-LacZ/E3N-Pnt-mt-LacZ*
***E3N-TCF-mt***: *w; E3N-TCF-mt-LacZ/E3N-TCF-mt-LacZ*

## Figure 5

B,B') *y, w; esg-LacZ/+; svb::GFP/+*

C) ***9CJ2***: *y, w; 9CJ2-LacZ/9CJ2-LacZ*
***9CJ2-Pdm-mt***: *y, w; 9CJ2-Pdm-mt-LacZ/9CJ2-Pdm-mt-LacZ*

D) ***control***: *esg-Gal4, UAS-GFP, tubP-Gal80*$^{ts}$*/+ ;*
***Svb***$^{REP}$: *esg-Gal4, UAS-GFP, tubP-Gal80*$^{ts}$*/+; UAS-Svb*$^{REP}$*/+*

E) ***control***: *esg-Gal4, UAS-GFP, tub-Gal80*$^{ts}$*/+; UAS-FLP, act>CD2>Gal4/+*
***Svb***$^{REP}$: *esg-Gal4, UAS-GFP, tub-Gal80*$^{ts}$*/+; UAS-FLP, act>CD2>Gal4/ UAS-Svb*$^{REP}$

F) ***control***: *esg-Gal4, UAS-mCD8::GFP/+; UAS-H2B::RFP, tubP-Gal80*$^{ts}$*/ +*
***Svb***$^{REP}$: *esg-Gal4, UAS-mCD8::GFP/+; UAS-H2B::RFP, tubP-Gal80*$^{ts}$*/ UAS-Svb*$^{REP}$

G) ***control***: *esg-Gal4, UAS-GFP, tubP-Gal80*$^{ts}$*/+;*
***Svb***$^{REP}$: *esg-Gal4, UAS-GFP, tubP-Gal80*$^{ts}$*/+; UAS-Svb*$^{REP}$*/+*

## Figure 6

A) ***control***: *esg-Gal4, UAS-GFP, tubP-Gal80*$^{ts}$*/+;*
***Notch-IR***: *esg-Gal4, UAS-mCD8::GFP, tubP-Gal80*$^{ts}$*; UAS-Notch*$^{RNAi}$*/+*
***Notch-IR+Svb***$^{REP}$: *esg-Gal4, UAS-mCD8::GFP, tubP-Gal80*$^{ts}$*; UAS-Notch*$^{RNAi}$*/UAS-Svb*$^{REP}$

B) ***control***: *esg-Gal4, UAS-GFP, tubP-Gal80*$^{ts}$*/+;*
***STAT-IR***: *esg-Gal4, UAS-mCD8::GFP, tubP-Gal80*$^{ts}$*/UAS-STAT92E*$^{RNAi}$*;*
***STAT-IR+Svb***$^{REP}$: *esg-Gal4, UAS-mCD8::GFP, tubP-Gal80*$^{ts}$*/UAS-STAT92E*$^{RNAi}$*; UAS-Svb*$^{REP}$ */+*

C) ***Wg***: *esg-Gal4, UAS-mCD8::GFP, tubP-Gal80*$^{ts}$*/UAS-Wg;*
***Wg+Svb***$^{ACT}$: *esg-Gal4, UAS-mCD8::GFP, tubP-Gal80*$^{ts}$ */UAS-Wg; UAS-OvoB/+*
***Wg+Svb***$^{REP}$: *esg-Gal4, UAS-mCD8::GFP, tubP-Gal80*$^{ts}$ *UAS-Wg; UAS-Svb*$^{REP}$ */+*
***Wg+Svb-IR***: *esg-Gal4, UAS-mCD8::GFP, tubP-Gal80*$^{ts}$*/UAS-Wg; UAS-svb*$^{RNAi}$*/+*

## Figure 7

A) ***control***: *MyoIA-Gal4, UAS-GFP, tubP-Gal80*$^{ts}$*/+;*
***svb-IR***: *MyoIA-Gal4, UAS-GFP, tubP-Gal80*$^{ts}$*/+; UAS-svb*$^{RNAi}$*/+*

| |
|---|
| ***Svb***$^{ACT}$: *MyoIA-Gal4, UAS-GFP, tubP-Gal80*$^{ts}$*/+; UAS-OvoB/+* |
| B) **control**: *MyoIA-Gal4, UAS-GFP, tubP-Gal80*$^{ts}$*/+;*<br>***svb-IR***: *MyoIA-Gal4, UAS-GFP, tubP-Gal80*$^{ts}$*/+; UAS-svb*$^{RNAi}$*/+*<br>***Svb***$^{ACT}$: *MyoIA-Gal4, UAS-GFP, tubP-Gal80*$^{ts}$*/+; UAS-OvoB/+* |
| C) **control**: *MyoIA-Gal4, UAS-GFP, tubP-Gal80*$^{ts}$*/+;*<br>***svb-IR***: *MyoIA-Gal4, UAS-GFP, tubP-Gal80*$^{ts}$*/+; UAS-svb*$^{RNAi}$*/+*<br>***Svb***$^{ACT}$: *MyoIA-Gal4, UAS-GFP, tubP-Gal80*$^{ts}$*/+; UAS-OvoB/+* |
| D) **control**: *MyoIA-Gal4, UAS-GFP, tubP-Gal80*$^{ts}$*/+;*<br>***svb-IR***: *MyoIA-Gal4, UAS-GFP, tubP-Gal80*$^{ts}$*/+; UAS-svb*$^{RNAi}$*/+*<br>***Svb***$^{ACT}$: *MyoIA-Gal4, UAS-GFP, tubP-Gal80*$^{ts}$*/+; UAS-Svb*$^{ACT::GFP}$ */+*<br>***Pri***: *MyoIA-Gal4, UAS-GFP, tubP-Gal80*$^{ts}$*/UAS-pri* |
| **Figure 8** |
| A) **control**: *MyoIA-Gal4, UAS-GFP, tubP-Gal80*$^{ts}$*/+;*<br>***Svb***$^{ACT}$: *MyoIA-Gal4, UAS-GFP, tubP-Gal80*$^{ts}$*/+; UAS-Svb*$^{ACT::GFP}$ */+* |
| B) **control**: *MyoIA-Gal4, UAS-GFP, tubP-Gal80*$^{ts}$*/+;*<br>***Svb***$^{ACT}$: *MyoIA-Gal4, UAS-GFP, tubP-Gal80*$^{ts}$*/+; UAS-Svb*$^{ACT::GFP}$ */+*<br>***Svb+pri***: *Myo-Gal4, UAS-GFP, tubP-Gal80*$^{ts}$*/UAS-pri; UAS-Svb*$^{REP}$ |
| C) **Svb**$^{ACT}$**:** *esg-Gal4, UAS-mDC8-GFP, tubP-Gal80*$^{ts}$*/UAS-Svb*$^{ACT::GFP}$ |
| **Figure EV1** |
| A) ***Enh-E6-lacZ***: *y, w ; ; E6-LacZ/E6/lacZ* |
| B) **control**: *esg-Gal4, UAS-YFP/+; Su(H)-GBE-Gal80, tubP-Gal80*$^{ts}$*/+*<br>***svb-IR***: *esg-Gal4, UAS-YFP/+; Su(H)-GBE-Gal80, tubP-Gal80*$^{ts}$*/UAS-svb*$^{RNAi}$ |
| C) **control**: *Su(H)GBE-Gal4, UAS-GFP/+; tubP-Gal80*$^{ts}$*/+*<br>***svb-IR***: *Su(H)GBE-Gal4, UAS-GFP/+; tubP-Gal80*$^{ts}$*/UAS-svb*$^{RNAi}$ |
| D) **control**: *tubP-Gal80*$^{ts}$*/+; Voila-GAL4, UAS-GFP.nls/+*<br>***svb-IR***: *tubP-Gal80*$^{ts}$*/+; Voila-GAL4, UAS-GFP.nls/UAS-svb*$^{RNAi}$ |
| E) **control**: *hs-FLP/+; actin<y+<Gal4/+; UAS–GFP/+*<br>***svb-IR***: *hs-FLP/+; actin<y+<Gal4/+; UAS–GFP/ UAS-svb*$^{RNAi}$ |
| F) **control**: *esg-Gal4, UAS-mCD8::GFP/+; UAS-H2B::RFP, tubP-Gal80*$^{ts}$*/+*<br>***svb-IR***: *esg-Gal4, UAS-mCD8::GFP/+; UAS-H2B::RFP, tubP-Gal80*$^{ts}$*/UAS-svb*$^{RNAi}$ |
| **Figure EV2** |
| A) **control**: *esg-Gal4, UAS-YFP/+; Su(H)-GBE-Gal80, tubP-Gal80*$^{ts}$*/+*<br>***pri-IR***: *esg-Gal4, UAS-YFP/+; Su(H)-GBE-Gal80, tubP-Gal80*$^{ts}$*/UAS-pri*$^{RNAi}$ |
| B) **control**: *esg-Gal4, UAS-mCD8::GFP, tubP-Gal80*$^{ts}$*/+ ;*<br>***EcR-IR***$^{\#1}$: *esg-Gal4, UAS-mCD8::GFP, tubP-Gal80*$^{ts}$*/UAS-EcR*$^{RNAi,BL\#58286}$<br>***EcR-IR***$^{\#2}$: *esg-Gal4, UAS-mCD8::GFP, tubP-Gal80*$^{ts}$*/+; UAS-EcR*$^{RNAi,\ BL\#29374}$*/+* |
| C) **control**: *esg-Gal4, UAS-YFP/+; Su(H)-GBE-Gal80, tubP-Gal80*$^{ts}$*/+*<br>***Pri***: *esg-Gal4, UAS-YFP/+i; Su(H)-GBE-Gal80, tubP-Gal80*$^{ts}$*/UAS-pri*<br>***EcR-DN***: *esg-Gal4, UAS-YFP /UAS-EcRDN*$^{B2w650A}$*; Su(H)-GBE-Gal80; tubP-Gal80*$^{ts}$*/+*<br>***EcR-DN+Pri***: *esg-Gal4, UAS-YFP /UAS-EcRDN*$^{B2w650A}$*; Su(H)-GBE-Gal80; tubP-Gal80*$^{ts}$*/UAS-pri* |
| D) **control**: *esg-Gal4, UAS-YFP/+; Su(H)-GBE-Gal80, tubP-Gal80*$^{ts}$*/+*<br>***Ubr3-IR***: *esg-Gal4 , UAS-YFP /UAS-Ubr3*$^{RNAi}$*; Su(H)-GBE-Gal80, tubP-Gal80*$^{ts}$*/+*<br>***Ubr3-IR+Svb***$^{ACT}$: *esg-Gal4, UAS-YFP / UAS-Ubr3*$^{RNAi}$*; Su(H)-GBE-Gal80, tubP-Gal80*$^{ts}$*/UAS-OvoB*<br>***Svb***$^{ACT}$: *esg-Gal4, UAS-YFP / UAS-Ubr3*$^{RNAi}$*; Su(H)-GBE-Gal80, tubP-Gal80*$^{ts}$*/UAS-OvoB* |

| |
|---|
| **Figure EV3** |
| A) ***control***: *esg-Gal4, UAS-mCD8::GFP, tubP-Gal80*$^{ts}$*/+ ;*<br>***Svb***$^{ACT(OvoB)}$: *esg-Gal4, UAS-mCD8::GFP, tubP-Gal80*$^{ts}$*/UAS-OvoB*<br>***Svb+Pri***: *esg-Gal4, UAS-mCD8::GFP, tubP-Gal80*$^{ts}$*/UAS-pri; UAS-Svb*$^{REP}$*/+* |
| A'') ***control***: *esg-Gal4, UAS-mCD8::GFP, tubP-Gal80*$^{ts}$*/+ ;*<br>***SvbACT***: *esg-Gal4, UAS-mCD8::GFP, tubP-Gal80*$^{ts}$*/UAS-Svb*$^{ACT::GFP}$ |
| B) ***control***: *esg-Gal4, UAS-YFP/+; Su(H)-GBE-Gal80, tubP-Gal80*$^{ts}$*/+*<br>***Svb***$^{ACT(OvoB)}$ : *esg-Gal4, UAS-YFP/+; Su(H)-GBE-Gal80, tubP-Gal80*$^{ts}$*/UAS-OvoB*<br>***Pri***: *esg-Gal4, UAS-YFP/UAS-pri ; Su(H)-GBE-Gal80, tubP-Gal80*$^{ts}$*/+*<br>***Svb+Pri***: *esg-Gal4, UAS-YFP /UAS-pri; Su(H)-GBE-Gal80, tubP-Gal80*$^{ts}$*/UAS-Svb*$^{REP}$ |
| C) ***snE1-wt***: *y, w; snE1-LacZ/snE1-LacZ*<br>***snE1-Svb-mt***: *y, w; snE1-Svb-mt-LacZ/snE1-Svb-mt-LacZ* |
| **Figure EV4** |
| B) ***E3N-wt***: *w; E3N-LacZ/E3N-LacZ*<br>***E3N-Pnt-mt***: *w; E3N-Pnt-mt-LacZ/E3N-Pnt-mt-LacZ*<br>***E3N-TCF-mt***: *w; E3N-TCF-mt-LacZ/E3N-TCF-mt-LacZ* |
| C) ***wild-type:*** *w;;*<br>***svb:*** *w, btd*$^{1}$*, svb*$^{1}$*/Y ;;*<br>***svb+E3N-wt::svb***: *w, btd*$^{1}$*, svb*$^{1}$*/Y; ; E3N-svbP-svb-cDNA::GFP/+*<br>***svb+E3N-TCF-mt::svb***: *w, btd*$^{1}$*, svb*$^{1}$*/Y; ; E3N-TCT-mt-svbP-svb-cDNA::GFP/+* |
| E) ***9CJ2-wt***: *y, w; 9CJ2-LacZ/9CJ2-LacZ*<br>***9CJ2-PDM-mt***: *y, w; 9CJ2-Pdm-mt-LacZ/9CJ2-Pdm-mt-LacZ* |
| **Figure EV5** |
| A) ***control***: *MyoIA-Gal4, UAS-GFP, tubP-Gal80*$^{ts}$*/+;*<br>***svb-IR***: *MyoIA-Gal4, UAS-GFP, tubP-Gal80*$^{ts}$*/+; UAS-svb*$^{RNAi}$*/+*<br>***Svb***$^{REP}$: *MyoIA-Gal4, UAS-GFP, tubP-Gal80*$^{ts}$*/+; UAS-Svb*$^{REP}$*/+*<br>***Ubr3-IR:*** *MyoIA-Gal4, UAS-GFP, tubP-Gal80*$^{ts}$*/+; UAS-Ubr3*$^{RNAi}$*/+* |
| B) ***control***: *MyoIA-Gal4, UAS-GFP, tubP-Gal80*$^{ts}$*/+;*<br>***Svb***$^{REP}$: *Myo-Gal4, UAS-GFP, tubP-Gal80*$^{ts}$*/+; UAS-Svb*$^{REP}$*/+* |
