# Supplementary material for: Steroid‐dependent switch of OvoL/Shavenbaby controls self‐renewal versus differentiation of intestinal stem cells
Source: EMBO J. 2020 Dec 29;40(4):e104347. doi: 10.15252/embj.2019104347 (PMC7883054; doi:10.15252/embj.2019104347)
Supplement: Supplementary file 2 — Expanded View Figures PDF [file EMBJ-40-e104347-s002.pdf]

Expanded View Figures

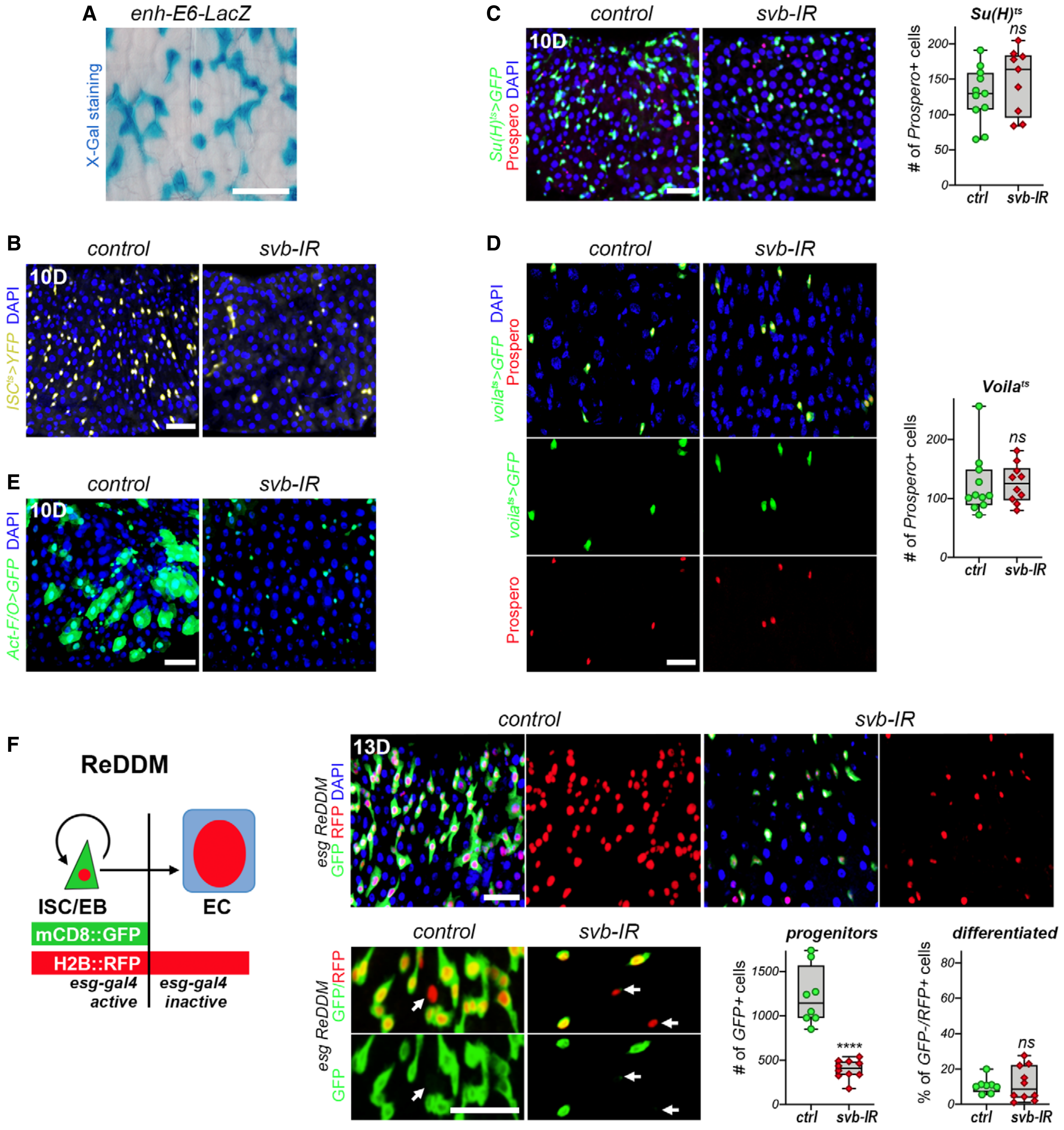

Figure EV1.

 © 2021 The Authors

Figure EV1. *svb* is required in ISCs/EBs and *svb* loss does not affect differentiation.

A Expression of *E6 svb* enhancer in the posterior midgut, as monitored by X-Gal staining of *E6-LacZ* reporter line.

B *ISC*^{ts} midguts expressing YFP alone (control), or expressing *svb*-RNAi. Samples were stained for YFP (yellow) and DAPI (blue); the quantification is shown in Fig 1E.

C *Su(H)*^{ts} midguts expressing GFP alone (control), or expressing *svb*-RNAi. Samples were stained for GFP (green), Prospero (red), and DAPI (blue). The graph shows quantification of the number of Prospero-positive cells (EEs). See also Fig 1E.

D *Voila*^{ts} midguts expressing GFP alone (control), or expressing *svb*-RNAi. Samples were stained for GFP (green), Prospero (red), and DAPI (blue); lower panels show separate channels. The graph displays quantification of the average number of Prospero-positive cells (EEs) in control conditions, or upon *svb*-RNAi treatment.

E *Act*^{ts}*F/O* midguts expressing GFP alone (control), or expressing *svb*-RNAi. Samples were stained for GFP (green) and DAPI (blue).

F Schematic representation of the ReDDM lineage tracing system (Antonello *et al*, 2015) in which *esg*^{ts} drives expression of both mCD8::GFP (green) and H2B::RFP (red). *esg*$^{+}$ cells are labeled by cytoplasmic GFP and nuclear RFP, while cells of their differentiated progeny only maintain the very stable H2B::RFP. Pictures show posterior midguts in control conditions, or upon expression of *svb*-RNAi. Samples were stained for GFP (green), RFP (red), and DAPI (blue). For each genotype, merge picture is show at left and the red channel at right. Bottom panels show magnified views; arrow show differentiated cells (RFP-positive/GFP-negative). Graphs show quantification of the number of GFP-positive (precursors) and the percentage of GFP-negative RFP-positive (differentiated) cells.

Data information: Boxes extend from the 25th to 75th percentiles, whiskers from min to max, the line in each box is plotted at the median; data were collected from three independent replicates. *P* values from Mann–Whitney tests are ns > 0.05, **** < 0.0001. Scale bars are 20 µm.

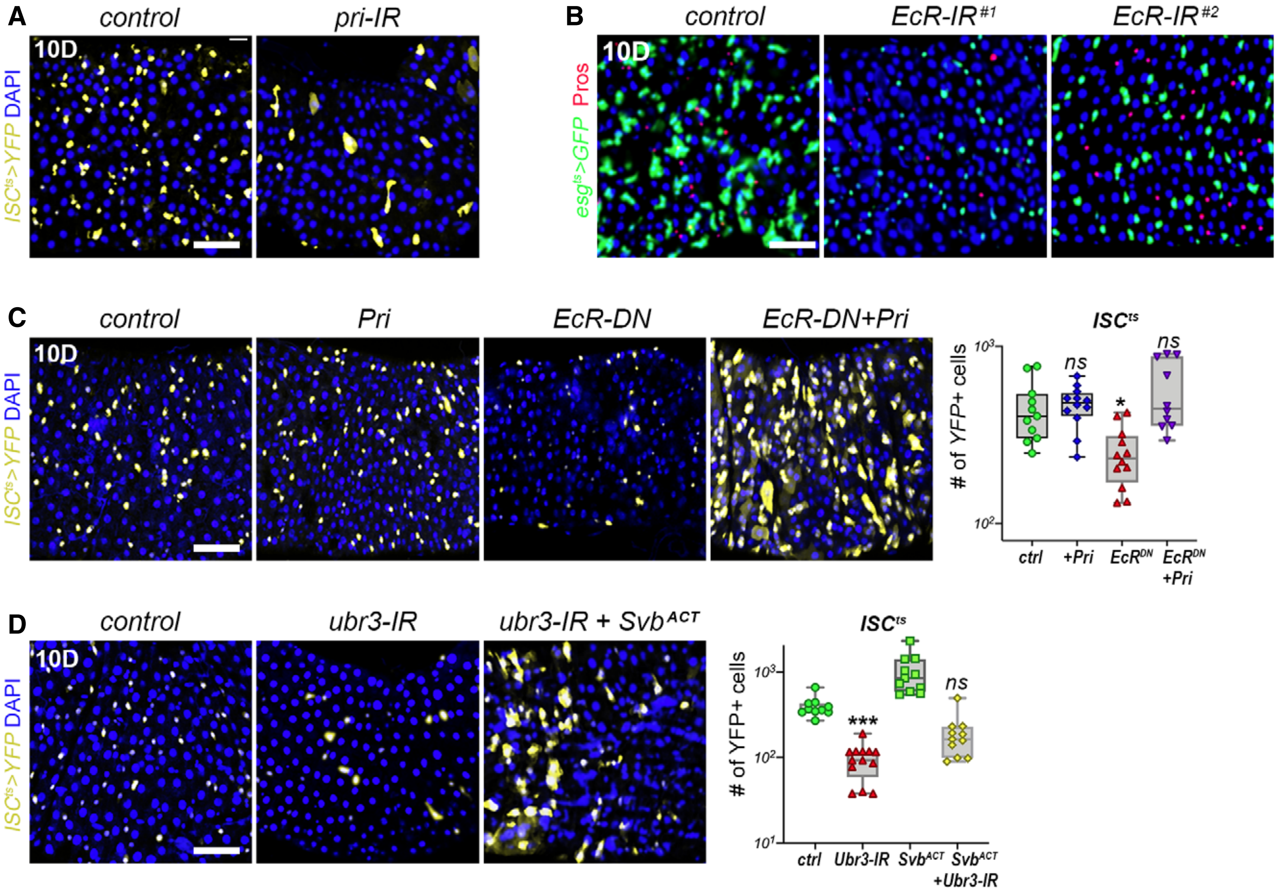

Figure EV2. Pri and Ubr3 are required for the maintenance of progenitor cells.

A *ISC*^{ts} midguts expressing YFP alone (control), or expressing *pri*-RNAi. Samples were stained for YFP (yellow) and DAPI (blue); quantification is shown in Fig 1E.

B *esg*^{ts} midguts expressing GFP alone (control), or expressing two RNAi lines that target non-overlapping regions of the Ecdysone receptor (EcR) mRNA. Samples were stained for GFP (green), Prospero (red), and DAPI (blue); quantification is shown in Fig 2E.

C *ISC*^{ts} midguts expressing YFP alone (control), or expressing UAS-*pri*, EcR-DN, and EcR-DN+ *pri*. Samples were stained for YFP (yellow) and DAPI (blue). The graph shows quantification of the number of YFP-positive cells for each genotype.

D *ISC*^{ts} midguts expressing YFP alone (control), or expressing *Ubr3*-RNAi, and *Ubr3*-RNAi+ OvoB. Samples were stained for YFP (yellow) and DAPI (blue). The graph shows quantification of the number of YFP-positive cells for each genotype.

Data information: Boxes extend from the 25th to 75th percentiles, whiskers from min to max, the line in each box is plotted at the median; data were collected from three independent replicates. *P* values from one-way ANOVA are: ns > 0.05; * < 0.05, *** < 0.001. Graphs are drawn with a log (10) Y scale. Scale bars are 20 µm.

© 2021 The Authors

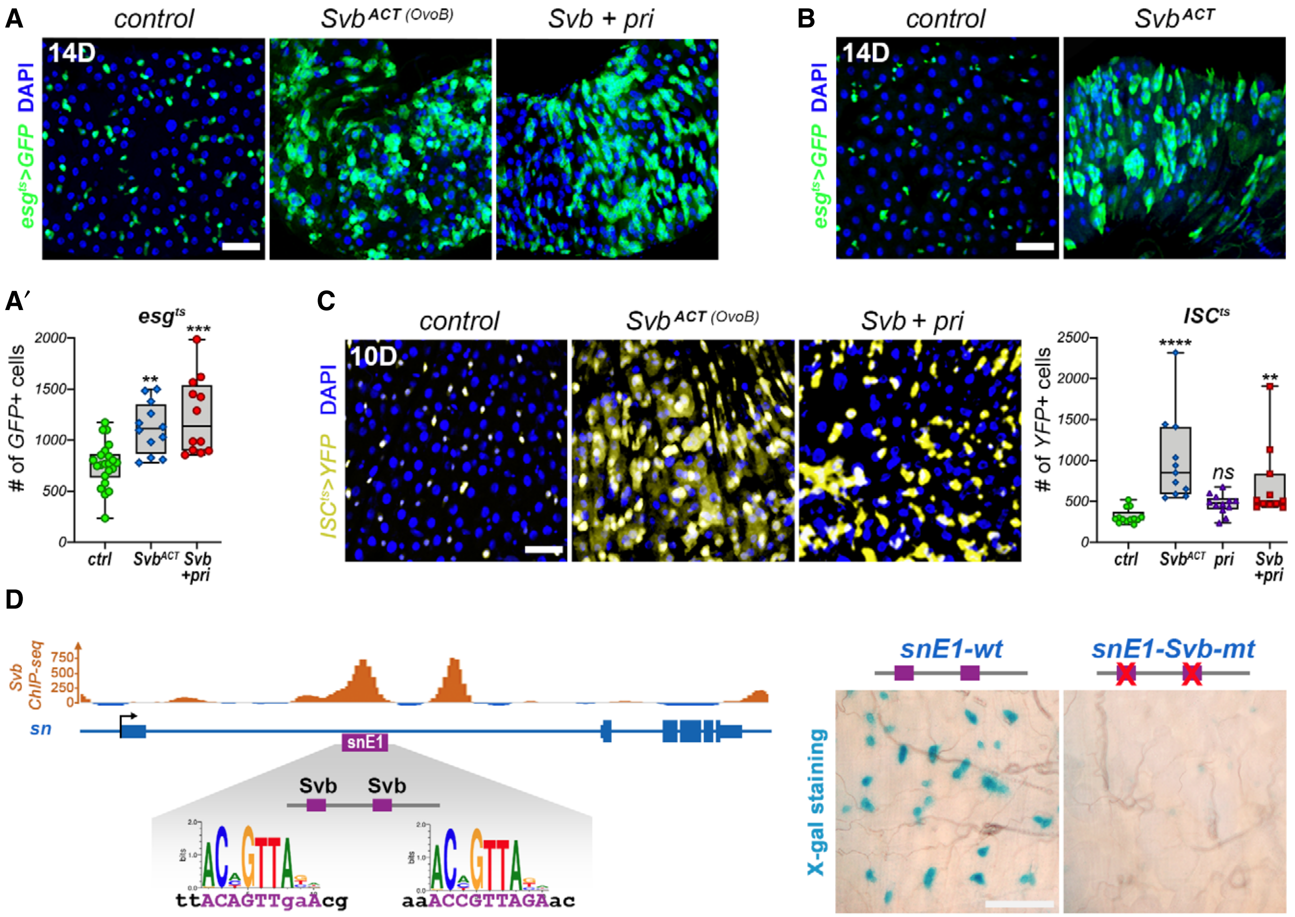

Figure EV3. Svb acts as a transcriptional activator in ISC/EB cells.

A, A′ *esg*^{ts} midguts expressing GFP alone (control), or expressing OvoB, and Svb^{REP}+ *pri*. Samples were stained for GFP (green) and DAPI (blue). The graph (A′) plots the number of GFP-positive cells in each genotype.

B *esg*^{ts} midguts expressing GFP alone (control) or expressing Svb^{ACT}. Samples were stained for GFP (green) and DAPI (blue).

C *ISC*^{ts} midguts expressing YFP alone (control), or expressing OvoB, and Svb^{REP}+ *pri*. Samples were stained for YFP (yellow) and DAPI (blue). The graph shows quantification of the number of YFP-positive cells in *ISC*^{ts} midguts expressing YFP alone (control), or expressing OvoB, Svb^{REP}+ *pri*, and *pri.*

D Snapshot view of ChIPseq signal in embryonic cells (Menoret *et al*, 2013), showing *in vivo* binding of Svb on the *singed* (*sn*) locus that encodes Fascin. The *snE1* enhancer (purple) contains two Svb-binding sites and is directly activated by Svb^{ACT}. Pictures at right show expression in the posterior midgut of wild-type *snE1* (*snE1-wt*), and a variant of it that contains mutation of the two Svb-binding sites (*snE1-Svb-mt)*. Samples were stained for β-Gal activity (cyan blue).

Data information: Boxes extend from the 25th to 75th percentiles, whiskers from min to max, the horizontal line is plotted at the median; data were collected from three independent replicates. *P* values from one-way ANOVA are ns > 0.05, ** < 0.01; *** < 0.001; **** < 0.0001. Scale bars are 20 μm.

 © 2021 The Authors

Figure EV4. Identification of transcription factors required for the activity of *E3N* and *9CJ2 svb* enhancers in the embryo.

- A Drawing of the *E3N svb* enhancer, with position of putative binding sites for Pnt (red) and TCF (green) factors, and evolution of DNA sequences across *Drosophila* species. Nucleotides in red represent point mutations introduced to disrupt either Pnt- or TCF-binding sites.
- B Consequences of knocking out Pnt- or TCF-binding sites on expression of the *E3N svb* enhancer in the embryonic epidermis. Pictures show ventral views of stage-15 embryos. Scale bar is 50 μm.
- C Trichome rescue assays (Crocker *et al*, 2015) showing the influence of TCF-binding sites on *E3N* function. Picture show cuticle preparations of wild-type and *svb*-mutant embryos, focusing on the ventral region of A6 segments. *svb* mutants display strong reduction in the number of trichomes, remaining ones being highly abnormal. Consistent with its expression pattern, *E3N* driving *svb* cDNA (*E3N-wt::svb*) rescues formation of the anterior-most trichome row (arrow). Knocking out TCF-binding sites (*E3N-TCF-mt::svb*) disrupts rescuing ability of the *E3N* enhancer. The graph plots the number of trichomes in the anterior-most row. Boxes extend from the 25th to 75th percentiles, whiskers from min to max, the horizontal line in each box is plotted at the median; data were collected from three independent replicates. *P* values from one-way ANOVA are ns > 0.5, **** < 0.0001. Scale bar is 15 μm.
- D Drawing of the *9CJ2 svb* enhancer, with position of putative binding sites for Pdm-1 (orange) and evolutionary conservation of the DNA sequence. Nucleotides in red show mutations that have been introduced to disrupt Pdm-binding sites.
- E Consequences of knocking down Pdm-1-binding sites on expression of the *9CJ2 svb* enhancer in the embryonic epidermis. Pictures show lateral (left), ventral (middle), and dorsal (right) views of stage-15 embryos. Scale bar are 50 μm.

Data information: Dmel, *Drosophila melanogaster*; Dsim, *Drosophila simulans*; Dyac, *Drosophila yacuba*; Dere, *Drosophila erecta*; Dfic *Drosophila ficusphila*; Dtak, *Drosophila takahashii*; Dana, *Drosophila ananassae*.

© 2021 The Authors

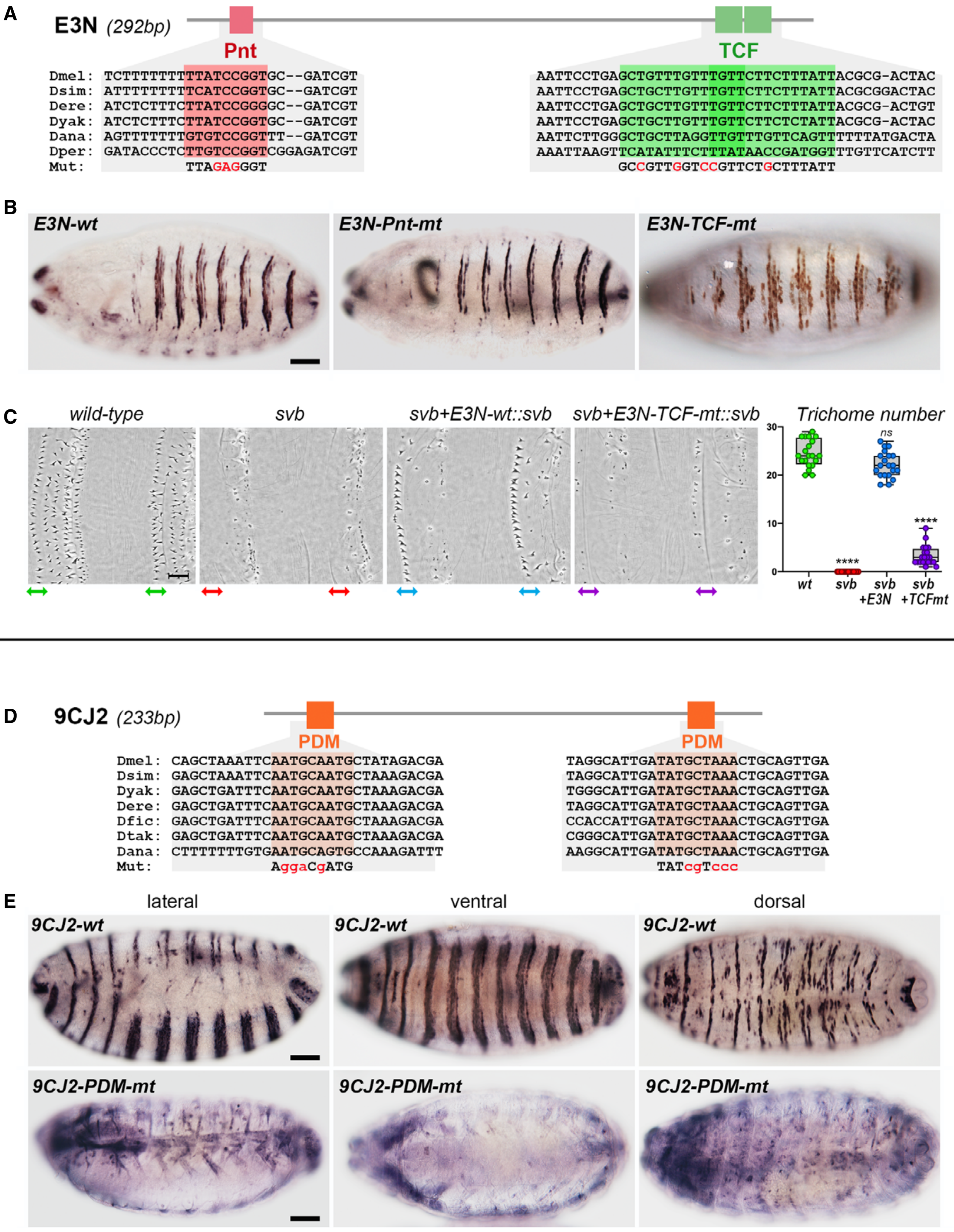

Figure EV4.

 © 2021 The Authors

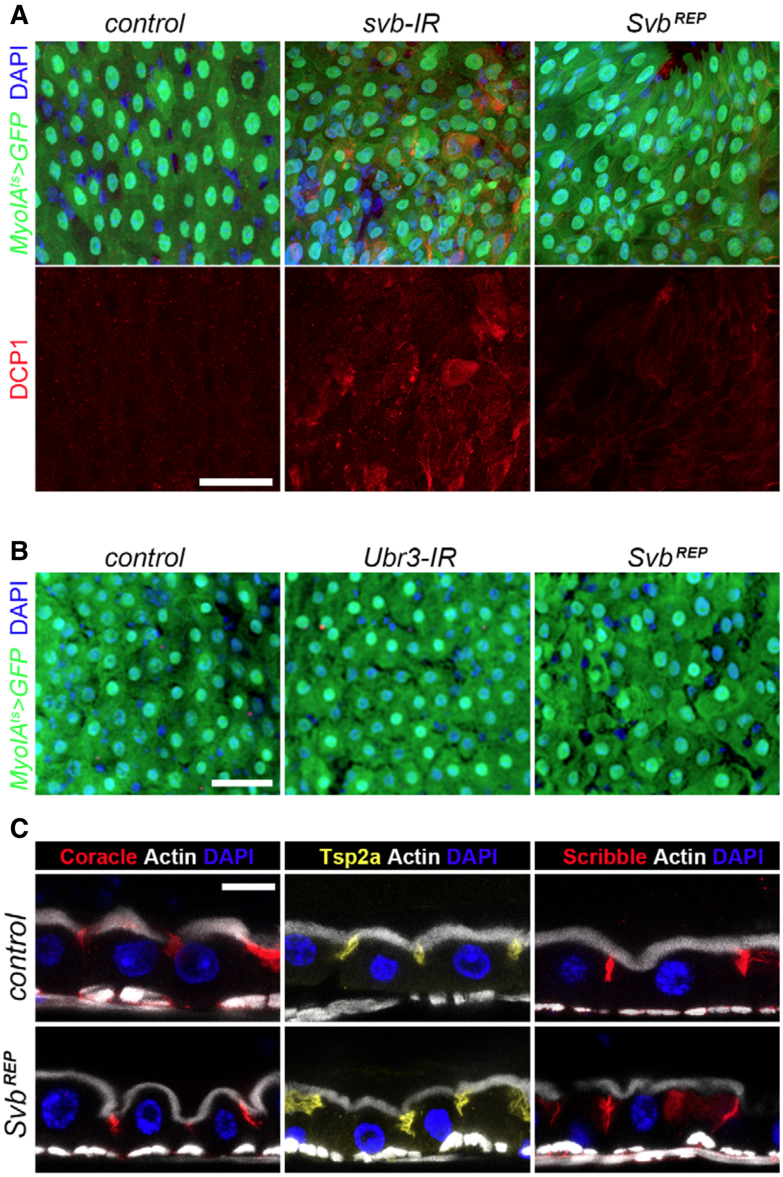

Figure EV5. Svb acts as a transcriptional repressor in differentiated enterocytes.

A *Myo1A*^{ts} midguts expressing GFP alone (control), or expressing *svb*-RNAi and Svb^{REP}. Samples were stained for GFP (green) and DAPI (blue). Lower panel shows staining for cleaved DCP1 (red). Scale bar is 20 μm.

B *Myo1A*^{ts} midguts expressing GFP alone (control), or expressing *Ubr3*-RNAi and Svb^{REP}. Samples were stained for GFP (green) and DAPI (blue). Scale bar is 20 μm.

C Cross sections of control *MyoIA*^{ts} midguts (expressing GFP and mCherry-RNAi, top row), or Svb^{REP} (bottom row). Samples were stained for F-actin (white), DAPI (blue), and Coracle (red), Tsp2a (yellow) or Scribble (red). Scale bar is 5 μm.

© 2021 The Authors
