# Supplementary figures and images for: Steroid‐dependent switch of OvoL/Shavenbaby controls self‐renewal versus differentiation of intestinal stem cells
Source: EMBO J. 2020 Dec 29;40(4):e104347. doi: 10.15252/embj.2019104347 (PMC7883054; doi:10.15252/embj.2019104347)

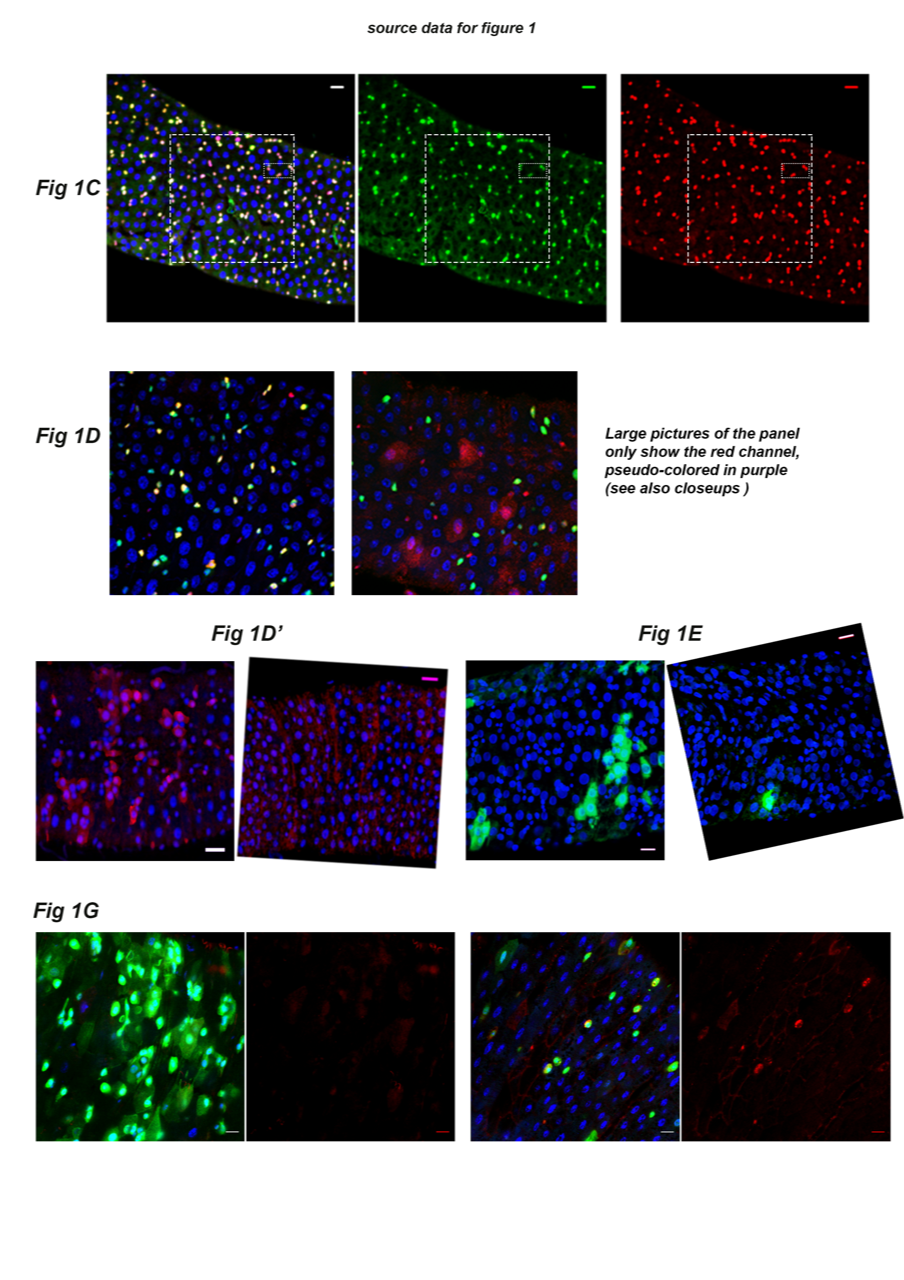

Supplement: Supplementary file 5 — Source Data for Figure 1 [file EMBJ-40-e104347-s004.zip › fig1-source-data/source_Figure1.png]

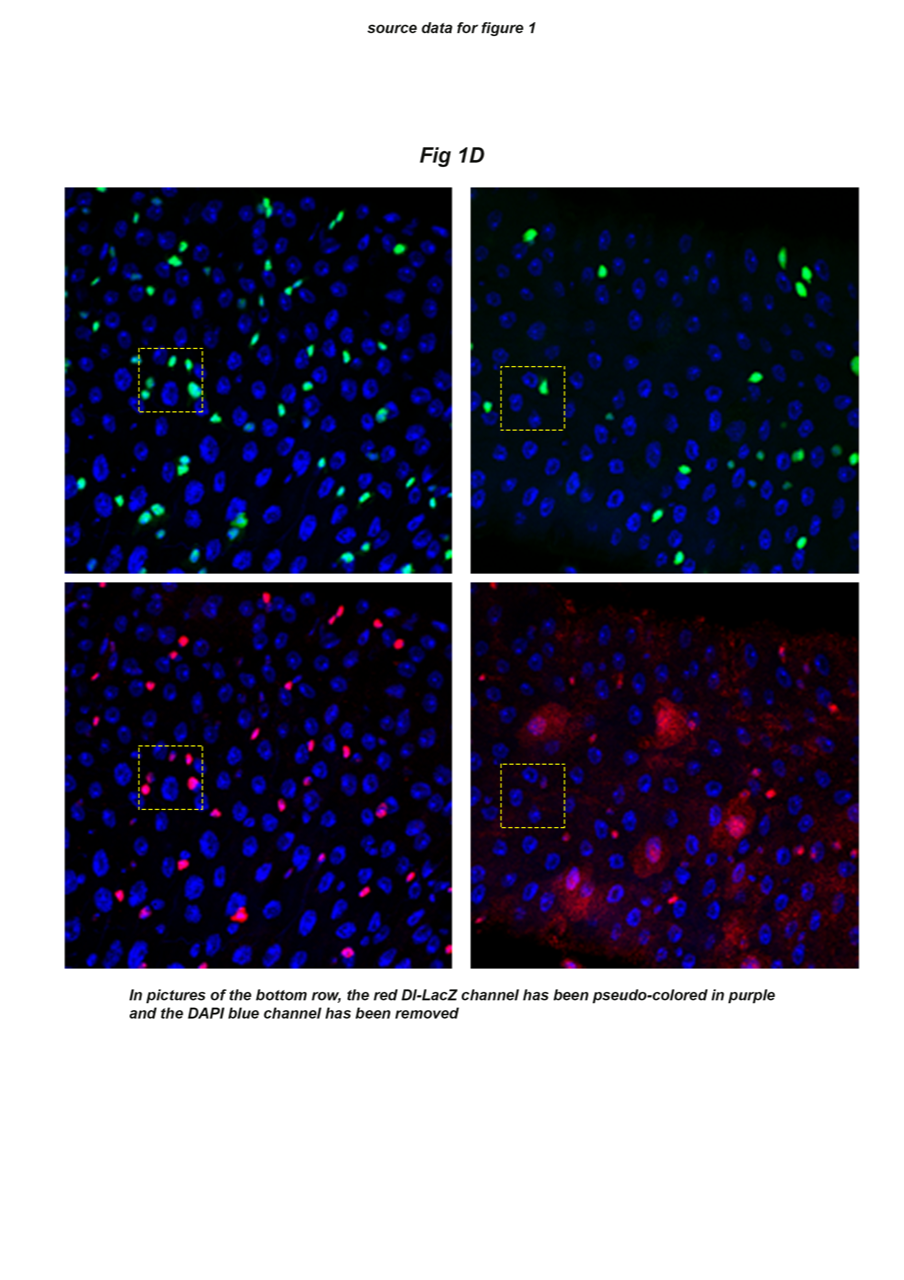

Supplement: Supplementary file 5 — Source Data for Figure 1 [file EMBJ-40-e104347-s004.zip › fig1-source-data/source_Figure1CloseUP.png]
